# A clinical prediction score for diagnosing unilateral primary Aldosteronism may not be generalizable

**DOI:** 10.1186/1472-6823-14-94

**Published:** 2014-12-11

**Authors:** Erik S Venos, Benny So, Valerian C Dias, Adrian Harvey, Janice L Pasieka, Gregory A Kline

**Affiliations:** Division of Endocrinology, Faculty of Medicine, University of Calgary, 1820 Richmond Road SW, Calgary, AB T2T 5C7 Canada; Department of Radiology, University of Calgary, Calgary, AB Canada; Division of Clinical Pathology, University of Calgary, Calgary, AB Canada; Department of Surgery, University of Calgary, Calgary, AB Canada

## Abstract

**Background:**

A published clinical prediction score indicated that a unilateral adrenal adenoma and either hypokalemia or an estimated glomerular filtration rate of 100 ml/min/1.73 m2 was 100% specific for unilateral primary aldosteronism. This study aimed to validate this score in a separate cohort of patients with primary aldosteronism.

**Methods:**

A review of patients with primary aldosteronism from June 2005 to July 2013 at a single center’s hypertension clinic. One hundred twelve patients with primary aldosteronism underwent successful adrenal vein sampling and the 110 patients with full data available were included in the final analysis. Adrenal vein sampling was performed all patients desiring surgery by the simultaneous collection of sample prior to and 15 minutes after a cosyntropin infusion with a 3:1 aldosterone/cortisol ratio diagnosing unilateral primary aldosteronism. The derived score was applied to the cohort. Sensitivity and specificity were calculated for clinical prediction score of ≥5 points.

**Results:**

There were 64 patients found to have unilateral primary aldosteronism and 48 had bilateral disease. A score ≥5 points had 64% sensitivity (95% confidence interval, 51–76) and 85% specificity (95% confidence interval, 71–94) for unilateral disease. Four patients had lateralization of primary aldosteronism to the side contralateral to the adenoma.

**Conclusions:**

The 100% specificity of the score for the unilateral origin of primary aldosteronism was not validated in this cohort with a score of ≥5 points. At best, a high score in this prediction rule may be an additional tool for helping to confirm a decision to offer patients adrenal vein sampling.

## Background

Primary aldosteronism (PA) is the most common cause of secondary hypertension with a 4-20% prevalence [1]. Using population-based screening, the underlying pathology is most frequently bilateral adrenal hyperplasia followed by unilateral aldosterone producing adenoma; less often, unilateral hyperplasia is also found [2]. Diagnosis involves screening patients with an aldosterone renin ratio (ARR) followed by a confirmatory test, if appropriate [3, 4]. Subsequently, computerized tomography (CT) of the adrenal glands can identify an adenoma as a source of aldosterone excess, though CT lacks sensitivity and specificity for unilateral PA [5]. It has been suggested that PA patients less than 40 years of age with a ≥1 cm unilateral adrenal adenoma could be directed to unilateral adrenalectomy without adrenal vein sampling (AVS) [5]. This approach applies to just 10% of patients with PA [6]. Those over 40 years of age with an adenoma or those with no imaging findings would be directed towards AVS to distinguish between unilateral and bilateral PA [3]. However, issues with AVS exist including lack of availability; technical procedural performance issues, especially when performed at lower volume centres; and possible risk of complications [7, 8].

A need exists, then, for a clinical prediction score (CPS) that predicts unilateral PA for a larger proportion of patients. A published CPS found 100% specificity for the identification of unilateral aldosterone excess in patients with confirmed PA in a retrospective analysis of patients 87 patients [6]. The predictive factors included an adrenal adenoma measuring ≥8 mm; hypokalemia (potassium ≤3.5 mmol/liter); and evidence of aldosterone-induced hyperfiltration with an increased estimated glomerular filtration rate (eGFR), using the modified diet in renal disease equation [9]. Each factor contributed a weighted number of points to the overall CPS with a maximum of 7 points for a patient with all three features (Table 1). A CPS of ≥5 was associated with 100% specificity for unilateral PA [6]. If this CPS were to be validated and the 100% specificity confirmed, practitioners would be able to confidently refer 30% of patients with PA and an imaged adenoma for surgical management without having AVS [6]. Two subsequent analysis of this CPS applied to different databases could not replicate the 100% specificity with performance characteristics of 38.8% specificity and 88.5% specificity [10] and sensitivity of 46% and a specificity of 80% [11] . The present study aimed to determine the validity of the CPS in a separate cohort of patients.

**Table 1:** **Clinical prediction score to predict lateralized AVS (ref.**[6])

Item	Points
Typical Conn’s adenoma on imaging	3
Hypokalemia <3.5 mmol/liter	2
eGFR (MDRD) (ml/min/1.73 m^2^)	
<80	0
80-100	1
>100	2
Total score	(max 7)

## Methods

### Patients

The study population was derived from the AVS database of a single, dedicated endocrine hypertension clinic with uniform screening for PA in all patients. Demographic, diagnostic and clinical outcome data from this cohort have been previously published [12], although the present study now includes updated patient data comprising June 2005 to July 2013. A single interventional radiologist performs the AVS, with success rates of 97% [13]. This database and its analysis have been approved by the University of Calgary Conjoint Health Research Ethics Board.

### Collected data and diagnostic criteria

At the initial consultation, clinical features systematically recorded included age, sex, body mass index, ethnicity, medications, and the measurement of blood pressure (BP) with the patients sitting quietly in a room after a 5-minute rest using the BpTRU measurement system (Coquitlam, BC, Canada).

All patients had measurement of ARR while seated in an upright position before 1000 h. Antihypertensive medications are not routinely stopped with the exception of mineralocorticoid antagonists, which are stopped for 6 weeks prior to testing [14]. The ARR is calculated from both lab values, using 0.1 ng/ml/h to avoid over-inflation of the ARR.

At our institution, an ARR greater than 550 (aldosterone in pmol/l and renin activity in ng/ml/hour; equivalent to cutoff of 15 when aldosterone expressed in ng/dl) has been used to identify patients with PA. Formal confirmatory testing has not been used in our unit since 2005; 90% of our current PA cohort are encompassed by an ARR of more than 4 times the upper limit of normal [4, 15] or a suppressed renin (less than 0.1 ng/ml/hr) while on an angiotensin converting enzyme inhibitor or an angiotensin receptor blocker [16]. There has not been a demonstrable increase in false positive PA diagnoses on pathology when our institution’s data has been reviewed [17].

Adrenal imaging and AVS are sequentially performed to characterize PA as either unilateral or bilateral. At our institution, AVS is only performed on PA patients who are considered to be surgical candidates and who desire surgery, consistent with a consensus published approach [18]. Thus, PA patients with complex, serious medical comorbidities are excluded from further investigation and this analysis. All surgical decisions are made based on AVS and imaging; no patients went to surgery without AVS being done. All patients selected for AVS had a 3 mm cuts CT scan of the adrenal glands with a classic adenoma defined as a nodule of 8 mm or greater and a normal contralateral adrenal contour [19]. Bilateral nodules, possible hyperplasia and nodules less than 8 mm were considered as lacking a classic adrenal adenoma. Hypokalemia was corrected using oral potassium supplements prior to AVS. Whenever possible, AVS was performed using non-dihydropyridine calcium channel blockers and alpha blockers for BP control although this was not always feasible due to hypertension concerns [20].

The AVS protocol as previously published included simultaneously collected baseline adrenal vein and inferior vena cava-cortisol and aldosterone samples followed by a 250 μg cosyntropin bolus and subsequent 25 ml infusion of 250 μg cosyntropin in 1000 ml D5W over 15 minutes [21] A second set of samples was then collected from each site. Adequate adrenal vein cannulation was defined as a selectivity index (adrenal/inferior vena cava-cortisol ratio) >3:1 for either baseline or stimulated results. Unilateral PA was diagnosed when the lateralization index (LI) from the dominant adrenal was > 3:1 compared to the non-dominant adrenal results on either the baseline or stimulated samples. In virtually all cases, lateralization was consistent on both baseline and stimulated samples [22].

Plasma aldosterone was measured by solid-phase I^125^ radioimmunoassay (RIA) using the Siemens Coat-A-Count® aldosterone assay (Siemens Healthcare Diagnostics, Tarrytown, NY). Mean interassay imprecision coefficients of variation were 13.0%, 6.8% and 6.8%, for aldosterone levels of 4.2 ng/dl, 24 ng/dl and 58.1 ng/dl, respectively. Dilutions were performed for the measurement of aldosterone levels greater than 72.2 ng/dl. Plasma renin activity (PRA) was measured by RIA of angiotensin I in the presence of reagents that inhibit angiotensin I-converting enzyme and angiotensinases using the GammaCoat® Plasma Renin Activity I^125^ RIA Kits (DiaSorin, Stilwater, MN). Mean inter-assay imprecision coefficients of variation were 11.1%, 7.8%, and 13.4% for PRA of 0.95 ng/ml/h, 5.17 ng/ml/h, and 21.2 ng/ml/h, respectively.

Plasma cortisol was measured by an automated electrochemiluminescence assay on the Roche Elecsys E170 (Roche Diagnostics, Mississauga, ON, Canada). Mean intra-assay imprecision coefficients of variation were 6.4%, 4.0%, and 3.2% for cortisol levels of 4 μg/dl, 22.6 μg/dl, and 32.9 μg/dl respectively. An initial dilution of 1:10 is auto-performed by the on-board instrument algorithm. When necessary a further off-line 1:25, 1:50 or 1:100 is selected and performed using manufacturers’ universal diluent to obtain an end-point level which is reported.

Patients with bilateral AVS results under the age of 40 undergo a dexamethasone suppression test to screen for glucocorticoid-remediable aldosteronism; suppression of plasma aldosterone by >80% determines who is referred for formal genetic testing [23]. From September 2009 until present, serum creatinine was measured within one week prior to AVS. Previously, it was measured within 6 months of AVS. Surgical candidates (unilateral PA) who agreed to surgery were referred for adrenalectomy. Postoperatively, the patients returned to the endocrine hypertension clinic for post-operative ARR measures and clinical reassessment, titrating any residual antihypertensive medications as needed. In general, patients in the clinic are offered follow-up visits at 4- to 6-week intervals during this period. Hypertension cure was defined as a BP of less than 140/90 mm Hg on no anti-hypertensive medications. Marked clinical improvement was defined as control of BP to less than 140/90 mmHg on fewer antihypertensive medications than at presentation.

### Statistical analysis

Unilateral and bilateral PA groups were defined using standard descriptive statistics, reporting group medians and intraquartile ratios (IQRs). Between-group continuous variables were analyzed using a two-tailed Mann–Whitney *U* Test for non-parametrically distributed data. Categorical data was compared by Chi-squared test with alpha set at 0.05. Each subject had a CPS calculated retrospectively, based upon the CPS of Küpers et al. [6]. Those with a CPS of ≥5 out of 7 were examined to determine whether the actual AVS results and outcomes confirmed unilateral PA. A receiver operating characteristic (ROC) curve was calculated to determine the overall sensitivity and specificity of the predictive rule to predict unilateral PA with special attention to the cut-off of ≥ 5 points. A separate analysis applied this CPS to those with a classic unilateral adenoma, as this group of patients would be the only group able to obtain a CPS ≥5 and where clinicians would have to consider whether or not to perform AVS prior to surgery. All computations were performed using Analyse-it software, 2011, (Analyse-it Software, Leeds, UK) except ROC curve analysis which was done with MedCalc 12.7 (MedCalc Software, Mariakerke, Belgium).

## Results

### Patients

**Figure 1:**
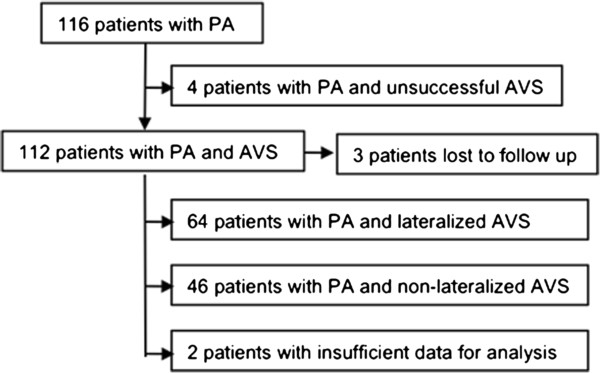
**Patient selection and distribution.**

The cohort consisted of 116 patients with PA. AVS was successful in 112 patients (96.5%), and full data exist for 110 of these 112 patients (Figure 1). Characteristics of the AVS cohort are presented in Table 2. An ARR was greater than 4 times the upper limit of normal in 78 (71%) of 110 patients [15]. Of the remaining 32 subjects, 23 had an elevated ARR while an ACE inhibitor or an angiotensin receptor blocker was being used for blood pressure control [16]. Unilateral PA was diagnosed by AVS in 64 patients (58%) and 53 elected ultimately to have surgical therapy. On surgical pathology, 34 had discrete adrenocortical adenoma, 14 had discrete adenoma with surrounding adrenal hyperplasia, and 5 had pure unilateral adrenal hyperplasia. Of the 37 patients who underwent postoperative ARR, 36 had an undetectable PAC or a normal ratio (ARR <550). The one patient with a persistently high post-operative ARR had an adrenocortical adenoma on pathology while also having a nodule on the contralateral adrenal gland, presumably representing a second aldosterone producing adenoma and an initial false positive AVS. Excluding the 3 patients lost to long term follow-up, 23 patients (44%) achieved clinical hypertension cure and sustained BP control of less than 140/90 mmHg and 28 patients (54%) achieved a marked improvement with a reduction of medications and control of BP < 140/90 mmHg.

**Table 2:** **Baseline characteristics of included patients**

	Lateralized AVS	Nonlateralized AVS	***P*** value
n	64	46	
Age, years	49 [40,58]	48 [39,55]	0.69
BMI (kg/m^2^)	29.0 [25.7,33.8]	30.9 [25.5,33.7]	0.89
Percentage of male subjects (%)	39 (61%)	20 (44%)	0.11
Systolic BP at presentation (mm Hg)	149 [136,161]	145 [131,158]	0.55
Diastolic BP at presentation (mm Hg)	90 [81,100]	91 [80,100]	0.90
Initial plasma aldosterone (ng/dl)	19.0 [14.7,29.4]	13.9 [9.0,17.7]	0.002
Aldosterone renin ratio (normal <15)	128 [47,266]	104 [50,142]	0.10
Plasma renin activity (ng/mL/h)	0.14 [0.1,0.32]	0.11 [0.1,0.25]	0.38
Initial serum K^+^ (mmmol/liter)	2.9 [2.6,3.5]	3.7 [3.2,3.8]	<0.0001
Serum potassium < 3.5 mmol/liter (%) mmmmmmolmmol/liter (%)	49 (77)	22 (46)	0.002
eGFR (ml/min/1.73 m^2^)	87.1 [67.8,96.5]	80.5 [67.9,98.3]	0.73
Adenoma ≥8 mm on CT (%)	46 (72)	16 (36)	<0.0001

Reported values represent number (percentage) or median [p25, p75]. n, Number of observations.

Mean clinical observation post AVS was 17.4 (IQR 1–60) months for the entire population and 8.9 months (IQR 1–49) post-operatively for those having surgery. The non-lateralized group had a median LI pre-cosyntropin stimulation of 1.50 (IQR 1.2, 2.4) and post-cosyntropin stimulation of 1.3 (IQR 1.1-1.6) at baseline and post-cosyntropin infusion. The lateralized group had a median LI (LI) of 11.8 (IQR 4.2-23.5) and 12.3 (IQR 5.5-30.8) in comparison. When patients with unilateral PA were compared with those who had bilateral PA, the unilateral patients had higher initial aldosterone, lower initial serum potassium, and higher proportion of unilateral classic adenoma on CT imaging (all p < 0.01).

### Retrospective application of the clinical prediction score to all patients

**Figure 2:**
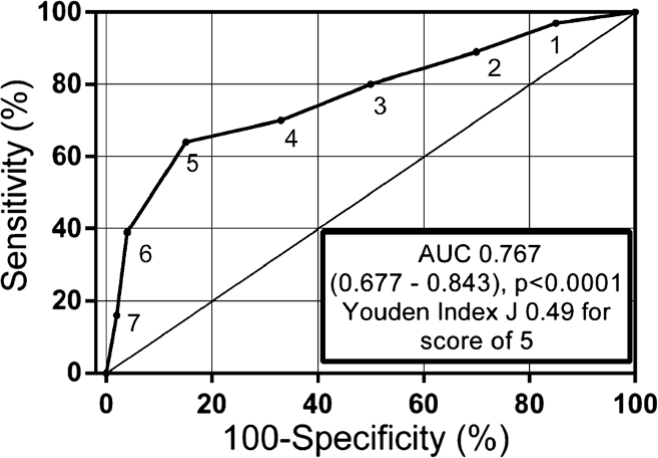
**ROC curve of the clinical prediction score from validation cohort.**

In our database, of 48 patients with a CPS of ≥5, 7 failed to show lateralization with AVS (Table 3). Thus, the CPS of ≥5 had a sensitivity of 64% (95% CI 51–76) and a specificity of 85% (95% CI 71–94). The positive predictive value was 85%. A CPS of ≥6 had a sensitivity of 39% (95% CI 27–52) and a specificity of 96% (95% CI 85–100). The ROC curve is shown in Figure 2. The area under the ROC curve was 0.77 (95% CI 0.68-0.84). Of the 7 patients with CPS ≥5 who did not show lateralization on AVS, all had a classic adenoma (mean size 19 mm) and all had hypokalemia. Six were over 40 years of age. The mean AVS baseline LI was 2.1 while the mean post-cosyntropin LI was 1.7. The single patient from this subset who was younger than 40 years old had a LI of 4.5 at baseline, though this decreased to 1.5 post-cosyntropin stimulation. We cannot exclude the possibility that this single subject actually does have unilateral PA, masked by ACTH use during AVS [24].

**Table 3:** **2×2 table using clinical prediction score of ≥5, applied retrospectively to the derivation cohort**

	Lateralized AVS	Nonlateralized AVS
Score ≥ 5	41 (64)	7 (15)
Score < 5	23 (36)	39 (85)
Total	64	46

Patients (%) reported.

In addition, of the 48 patients with a CPS ≥5, 4 patients had imaging showing an adrenal adenoma on the opposite adrenal from the side of AVS-defined unilaterality. Therefore, considering the 7 false positives by CPS and the 4 discordant positives, 11 of 78 (15%) of patients would have had unnecessary surgery or the wrong adrenal gland removed using this CPS. Of the 23 patients with unilateral PA by AVS, but a CPS <5 (Table 3), 14/23 had eukalemia, 22/23 had an eGFR less than 100 ml/min/m^2^, and 16/23 had no findings of classic adenoma of CT imaging, representing 36% of the entire unilateral PA group. When patients under 40 years of age with a unilateral adenoma (≥10 mm) were assessed for this criteria’s performance characteristics, it was found to have a sensitivity of 94% (95% CI 70–100) and a specificity of 75% (43–95), although this subanalysis only comprised 28 subjects. The 3 patients with an adrenal adenoma (11 and 13 mm) but a nonlateralized AVS all had an ARR above 4 times the upper limit of normal and 2 of 3 had hypokalemia. One of these patients was described above: baseline LI of 4.5 decreasing to 1.5 post-cosyntropin stimulation (possible lateralized PA, treated as nonlateralized on the basis of post cosyntropin testing LI < 3). The other two patients had baseline LIs of 2.7 and 1.7, and post-cosyntropin stimulation LIs of 2 and 1.2.

## Discussion

We aimed to determine the validity of the CPS developed by Küpers et al. in our own independent cohort of PA patients. With our validation cohort, the CPS of ≥5 had a sensitivity of 64% (95% CI 51–76) and a specificity of 85% (95% CI 71–94), all as defined by AVS but still not accounting for the few cases where imaging and AVS findings are discordant. This specificity of this CPS is similar to that reported previously, yet does not approach the 100% specificity that might be necessary for routine clinical use in surgical decision-making. With our total unilateral disease prevalence of 58%, this CPS had a positive predictive value of 85%. A CPS of ≥6 did perform better with a specificity (96%) approaching 100%. If this cut point were used, then the CPS would apply to approximately 20% of patients with PA. This point has not been previously emphasized; although few patients may achieve such a high score, for those who do, a near-perfect specificity may well add significantly to the clinical confidence in a lateralized diagnosis.

A further concern is demonstrated by 4 patients with a CPS of ≥5 who had AVS lateralization contralateral to the side of the adenoma, leading to a total of 11 apparently unilateral PA patients having surgery that would be unnecessary when one also includes the 7 clearly bilateral PA patients who also had a CPS ≥5. The derivation cohort had just one patient that had these characteristics [6], as did one of the validation cohorts [11]. These cases, while few, are a sobering caution that omission of the AVS step does carry a small risk of misclassification and inappropriate management. Interestingly, we also found less than 100% specificity (75%) for unilateral disease in patients under 40 who also had a typical adenoma of ≥10 mm, which is also contrary to the findings of the derivation cohort and others [5, 6, 11]. This may reflect differing biochemical definitions as to what constitutes “unilateral” AVS or a differing size definition of an adrenal nodule. Centers using more strict or more lenient lateralization indices may well show apparent differences in the proportions of patients deemed unilateral and verification will be limited to those who actually undergo adrenalectomy.

It is increasingly recognized that PA can have multiple different molecular etiologies including various genes and ectopic hormone receptors [25, 26]. It would therefore be expected that there may well be significant variation in disease presentation, diagnostic features and possibly response to therapy according to such differing causes and modified by variables that influence penetrance such as age, sex and ethnicity. In the future, more sophisticated molecular studies of confirmed PA cases may permit a better understanding of the apparent differences between PA study populations.

### Study strengths

The approach to diagnosis and therapy were uniform for the duration of the database. In addition, CT of the adrenal glands and AVS were performed on all patients who were considering surgery, which eliminates any potential bias due to selective use of AVS and ensured that the CPS could be applied to every patient in our database. Our centre has a very low rate of AVS failure ensuring broad inclusion of a high number of patients and the reliability of the procedure [12]. Finally, standardized longitudinal assessment of patient-level outcomes such as BP readings, number of medications, follow-up ARR after surgery, and surgical pathology confirmed a clinical biochemical diagnosis of PA in the cohort that underwent surgery [17].

### Study limitations

As will be true of all studies comparing a test performed at different endocrine units, differences between study cohorts always limit the generalization of any findings to other centres where patients and the diagnostic approaches can vary. We were able to analyze 110 patients in our cohort compared to 87 in the derivation cohort and 78 in the other validation cohort. Our cohort featured a high proportion of patients with unilateral PA (58%), which is a comparable proportion to the 56% in the derivation cohort. This higher proportion than in other populations likely reflects referral bias as well as reflects the practice where an increasing proportion of lateralized patients is found when AVS is performed routinely. As well, it is possible that routine clinical decision making in the unit is already selecting out patients with high risk features for further investigation.

Our diagnosis of PA is based on the presence of an elevated ARR ratio without the requirement of a confirmatory test prior to CT scanning and AVS and this certainly reflects a practice that differs from most other endocrine hypertension units. Other units may also measure ARR under different conditions and use different cut-off values for definition of PA. This diagnostic variation might well affect the final prevalence of unilateral PA in different centers [27, 28]. In our cohort, surgical management based on our diagnostic criteria lead to surgical cure or improvement of hypertension in all but one of our patients (thought to have bilateral APA based on post-operative biochemistry and imaging). Ideally all subjects should have had a post-operative ARR measured to confirm resolution of PA rather than depending upon blood pressure and pathology alone. While one could consider possible false positive PA case ascertainment, the diagnostic agreement between our approach and that of the derivation cohort was remarkably high (56% and 58% unilateral PA). Other units with a lower prevalence of unilateral PA might find very different specificity for the CPS.

The CT scans were read by the institution’s radiologists and not subjected to a blinded panel review. It is possible that a blinded, multi-reviewer panel could have come up with differing interpretations of the CT images which could affect the subject classification and score although the CPS performance could be either better or worse than estimated here. Our pragmatic use of CT scans as reported by a clinical radiologist may actually be an asset in that it reflects routine use of imaging data in a functional endocrine hypertension unit, in the same way that the CPS is intended to be used.

Compared to the derivation cohort, our cohort shared the finding of increased hypokalemia, increased aldosterone, and the higher proportion of patients with an adenoma in the lateralized AVS group compared to the non-lateralized AVS group. Unlike the derivation cohort, there were no differences in eGFR between groups. This could represent a type 2 error from small numbers. However, this would be unlikely to affect the specificity of the CPS as all patients who had a CPS of ≥5, but did not have a lateralized AVS had hypokalemia and an adenoma.

The clinical prediction score itself has several important limitations to widespread use, notably the use of GFR as a predictive value. A person with PA may have widely variable measures in GFR depending upon age, concomitant medication use and duration of aldosteronism, all of which can influence a single measure of GFR without reference to the laterality status. A second PA subtype prediction rule has also recently appeared in the literature [29] which purports to show that the combination of plasma aldosterone, potassium and post-captopril ARR may help predict unilaterality. However, this has also been generated using a small sample size and will also need external validation attempts. A multi-national standardized PA database is likely necessary to have adequate power for the discovery of all significant clinical factors that may ultimately form a more accurate and generalizable clinical prediction rule.

## Conclusions

Clinical prediction scores are an important tool in clinical decision making, though they require validation and an impact analysis to determine whether they should be applied broadly [30]. Despite the promise of this CPS to include a higher proportion of patients who could proceed directly to surgery without AVS, the specificity was not sufficiently high to exclude the possibility of an unnecessary surgery. The CPS performed less well when it was applied to those already known to have a unilateral adenoma on imaging as it would be used in clinical practice. Overall, 15% of patients would have unnecessary or unhelpful surgery if this CPS was applied and surgery undertaken without AVS. The CPS may be useful to help select patients that should indeed have AVS with a high probability of finding unilateral disease. Thus, this CPS in its current form may be complimentary to AVS rather than a replacement for AVS. Despite the limitations of AVS, for patients who desire surgical therapy of PA, AVS should still be undertaken to confirm surgical suitability and planning. Endocrine hypertension specialty units should consider local validation of the CPS before clinical implementation.

### Consent

Written informed consent was obtained from our patients, which include their clinical data for the publication of this report.

## Abbreviations

ARR: Aldosterone renin ratio
AVS: Adrenal vein sampling
BAH: Bilateral adrenal hyperplasia
BP: Blood pressure
CI: Confidence interval
CPS: Clinical prediction score
CT: Computed tomography
eGFR: Estimated glomerular filtration rate
LI: Lateralization index
PA: Primary aldosteronism
PAC: Plasma aldosterone concentration
RIA: Radioimmunoassay
ROC: Receiver operating characteristic.
